# Genomic Insights into Biofilm-Forming *Enterococcus faecalis* SK460 Isolated from a Chronic Diabetic Ulcer Patient

**DOI:** 10.1128/genomeA.01463-17

**Published:** 2018-01-11

**Authors:** Karthika Suryaletha, Lekshmi Narendrakumar, Joby John, Dinesh Reghunathan, Manoj Prasannakumar, Sabu Thomas

**Affiliations:** a Cholera and Biofilm Research Lab, Rajiv Gandhi Centre for Biotechnology, Trivandrum, Kerala, India; b Research Scholar, University of Kerala, Kerala, India; c Department of Surgery, Government Medical College Hospital, Trivandrum, Kerala, India; d Genomics Core Facility, Rajiv Gandhi Centre for Biotechnology, Trivandrum, Kerala, India

## Abstract

*Enterococcus faecalis* is recognized as one of the leading pathogens causing nosocomial infections. Here we report a draft genome sequence of *Enterococcus faecalis* SK460, isolated from a chronic diabetic foot ulcer patient. This strain exhibits various biofilm-associated genes, virulence genes, and antibiotic-resistance genes related to aminoglycoside, macrolide, and tetracycline resistance.

## GENOME ANNOUNCEMENT

*Enterococcus faecalis* is a major nosocomial pathogen causing serious ailments, including endocarditis, wound infections, urinary tract infections, and infections associated with indwelling medical devices (1). This pathogen is highly adapted to thrive under nutrient-deprived and stressful conditions (2) and is able to form robust biofilm on tissue surfaces as well as abiotic surfaces (3). The organism’s degree of pathogenicity can be attributed to its emerging antibiotic resistance and strong biofilm-forming ability (4). Although several putative virulence factors and cellular processes are involved in enterococcal biofilm formation (5), the knowledge regarding the molecular mechanisms underlying their regulation is sparse.

*E. faecalis* strain SK460 was isolated from wound swabs obtained from a patient with chronic diabetic foot ulcer from a tertiary care hospital in Trivandrum, Kerala, India. The strain was grown overnight at 37°C in brain heart infusion agar and was identified by 16S rRNA gene sequencing. The isolate showed beta-hemolytic activity, and antimicrobial susceptibility testing performed by disc diffusion assay revealed resistance to azithromycin, erythromycin, amikacin, kanamycin, gentamicin, streptomycin, tetracycline, and ciprofloxacin. Biofilm assessment using crystal violet assay and confocal laser scanning microscopy unveiled its high biofilm-forming potential.

Genomic DNA was extracted using the Wizard genomic DNA purification kit (Promega) according to the manufacturer’s instructions. The DNA quality was quantified using a NanoDrop spectrophotometer (Thermo Scientific, Waltham, MA, USA) and a Qubit version 2.0 fluorometer (Life Technologies, Carlsbad, CA, USA). DNA integrity was checked by agarose gel electrophoresis. The sequencing was carried out on an Ion Proton PGM using a 318 v2 chip. *De novo* assembly of the raw reads was done with the SPAdes version 3.1.0 assembly tool. The draft genome has 3,144,087 bp in a total of 196 contigs, with an *N*_50_ of 27,553 bp and GC content of 37.12%. The sequences were annotated using NCBI Prokaryotic Genome Annotation Pipeline version 4.2 and analyzed by the Rapid Annotations using Subsystems Technology (RAST) server. The annotation process detected a total of 3,405 genes, of which 2,066 are coding sequences (CDSs), 1,286 pseudogenes, 366 subsystems, and 53 RNAs, including 44 tRNA genes, 5 rRNA genes, and 4 noncoding RNAs (ncRNAs).

ResFinder detected three genes coding aminoglycoside resistance [*ant*(*6*)*-Ia*, *aac6-aph2*, and *aph3*′-*III*], two genes coding macrolide resistance (*ermB* and *lsaA*), and a tetracycline resistance gene (*tetM*). VirulenceFinder identified biofilm-associated genes and virulence genes, including endocarditis and biofilm-associated pili (*ebpA*,* ebpB*, and *epbC*), cytolysin (*cyl*), hyaluronidase (*hylA and hylB*), aggregation substance (*agg*), adhesion-associated genes (*ace* and *bopD*), gelatinase (*gelE*), enterococcal surface protein (*esp*), enterococcal endocarditis antigen from *E. faecalis* (EfaAfs), enterococcal leucine-rich protein A (ElrA), and colicin. PlasmidFinder identified a plasmid, rep9, which is similar to the *E. faecalis* V583 plasmid pTEF2. Genome sequencing of this biofilm-forming *E. faecalis* revealed a host of virulence factors that likely contribute to its pathogenicity, especially genes significant for biofilm formation. Comparative genomics and in-depth analysis will aid in revealing the molecular background sustaining the pathogenesis and biofilm mode of infection of this predominant nosocomial pathogen.

### Accession number(s).

This whole-genome shotgun project has been deposited at DDBJ/ENA/GenBank under the accession number NIXL00000000. The version described in this paper is the first version, NIXL01000000.
